# The puzzle of immune phenotypes of childhood asthma

**DOI:** 10.1186/s40348-016-0057-3

**Published:** 2016-07-28

**Authors:** Katja Landgraf-Rauf, Bettina Anselm, Bianca Schaub

**Affiliations:** 1 Department of Pulmonary and Allergy, Dr. von Hauner Children’s Hospital, LMU, Lindwurmstraße 4, 80337 Munich, Germany; 2 Member of German Lung Centre (DZL), CPC, Munich, Germany

**Keywords:** Asthma, Endotypes, Childhood asthma, Biomarkers, Immune mechanisms, Phenotypes, Puzzle

## Abstract

Asthma represents the most common chronic childhood disease worldwide. Whereas preschool children present with wheezing triggered by different factors (multitrigger and viral wheeze), clinical asthma manifestation in school children has previously been classified as allergic and non-allergic asthma. For both, the underlying immunological mechanisms are not yet understood in depth in children. Treatment is still prescribed regardless of underlying mechanisms, and children are not always treated successfully. This review summarizes recent key findings on the complex mechanisms of the development and manifestation of childhood asthma. Whereas traditional classification of childhood asthma is primarily based on clinical symptoms like wheezing and atopy, novel approaches to specify asthma phenotypes are under way and face challenges such as including the stability of phenotypes over time and transition into adulthood. Epidemiological studies enclose more information on the patient’s disease history and environmental influences. Latest studies define endotypes based on molecular and cellular mechanisms, for example defining risk and protective single nucleotide polymorphisms (SNPs) and new immune phenotypes, showing promising results. Also, regulatory T cells and recently discovered T helper cell subtypes such as Th9 and Th17 cells were shown to be important for the development of asthma. Innate lymphoid cells (ILC) could play a critical role in asthma patients as they produce different cytokines associated with asthma. Epigenetic findings showed different acetylation and methylation patterns for children with allergic and non-allergic asthma. On a posttranscriptional level, miRNAs are regulating factors identified to differ between asthma patients and healthy controls and also indicate differences within asthma phenotypes. Metabolomics is another exciting chapter important for endotyping asthmatic children. Despite the development of new biomarkers and the discovery of new immunological molecules, the complex puzzle of childhood asthma is still far from being completed. Addressing the current challenges of distinct clinical asthma and wheeze phenotypes, including their stability and underlying endotypes, involves addressing the interplay of innate and adaptive immune regulatory mechanisms in large, interdisciplinary cohorts.

## Introduction

Asthma is a complex chronic pulmonary disease with reversible airflow obstruction, which affects adults and children. The prevalence of asthma symptoms in 13- to 14-year-old children ranges between 5 and 20 % in different parts of the world with partly increasing trend [1]. Its pathogenesis is still not completely clear, despite the extensive research being conducted. Genetic background in addition to a variety of influences, including epigenetic factors and for example environmental exposure and infections among many others, are involved in its development [2]. Labeling all children suffering from asthma-suggestive pulmonary symptoms as “asthmatics” does not reflect how heterogeneous this group is in its clinical presentation, progress, and response to therapy [3, 4]. However, viewing asthma as a syndrome comprising different childhood wheeze phenotypes defined by distinct endotypes, including specific immunological patterns and environmental factors, is most likely more accurate [2, 5]. Currently, treatment guidelines are only beginning to integrate insights from research about underlying causes of this heterogeneity. Mostly, they mainly advise universal treatment steps in asthmatic children, resulting in similar medication for asthmatic patients differing primarily in varying priorities of steroids or leukotriene antagonists and drug doses [6, 7]. This way of treatment leaves a considerable 5–10 % of so-called non-responders, classified as uncontrolled or severe asthma in adults [8]. Classifying the disease into different, more specific clinical phenotypes and biological endotypes is the first step to a more individually tailored and effective therapy for children suffering from asthma [9].

## Course of different phenotypes

There are several tools to diagnose asthma early in life, such as the International Study of Asthma and Allergy in Childhood (ISAAC) criteria or the modified asthma predictive index (mAPI) [3, 10]. Defining clinical phenotypes for preschool and school children is a way to differentiate between groups of asthma patients presenting with similar combinations of symptoms [5]. The clinical approach includes the patient’s history, diagnostic techniques, and treatment responses. Preschool children with wheeze have been divided into two groups, namely multiple-trigger wheeze (MTW) and episodic viral wheeze (EVW), triggered commonly by infections [3, 11]. Data about prevalence and, even more important, about stability of these phenotypes have only been published recently. For example, van Wonderen et al. showed in a cohort study that stable MTW and EVW are relatively uncommon as about 80 % of the children changed phenotypes in an observation period of 24 months. However, children with stable MTW had a largely increased risk for development of childhood asthma [12]. In 2014, Brand et al. proposed some modifications to the recommendations made in 2008. Due to varying and changing patterns of wheeze phenotypes in young children over time and following treatment, they now proposed to rather focus on pattern, frequency, and severity of symptoms [7].

One of the most common parameters for classification at school-age is specific IgE, an immunoglobulin used to assess allergic sensitization as a mean of differentiation between allergic and non-allergic asthmatics. Positive specific IgE with characteristic clinical symptoms in addition to pulmonary symptoms is distinctive for allergic asthmatics while a low level and/or negative specific IgE without clinical symptoms is defined as non-allergic asthma phenotype [13]. However, as other non-allergenic factors such as viral infection and air pollution trigger the IgE pathway and/or influence the development of asthma, the real contribution of IgE-mediated allergy in asthma has been discussed. In accordance, recent studies about the use of IgE in prediction of response to omalizumab (an anti-IgE antibody) have shown that the value and specificity of IgE as a biomarker is increasingly unclear. Using the count of blood eosinophils, usually elevated in asthma, has shown more promising but still not satisfying results, suggesting the existence of subphenotypes [14]. A main problem of clinical phenotyping is that the definition is often biased, as mostly only the most dominant disease feature is used for classification [5].

Another way is a more objective, epidemiologic approach driven by data from birth cohorts. Mainly latent class analysis (LCA) is used to identify groups with similar features for a large, heterogeneous group of subjects [3, 4]. Epidemiologic phenotyping yields longitudinal time patterns, defining transient, and persistent or late-onset wheeze in children. However, this is of limited use for clinical management of the patient as LCA can only be performed in retrospective, and is more commonly used for research purposes. Studies merging the two methods to benefit from both show that in some cases, the phenotypes defined by LCA fit with the clinical phenotype, but in others, they differ greatly. Thus, there is a considerable number of children without a proper diagnosis and therefore without appropriate treatment [3, 4]. The challenge both methods face is dealing with changes in the clinical picture over time, as the assignment of a clinical phenotype tends to change. One option is that change is part of the phenotype, thus defining a trajectory for this kind of wheeze phenotype. Alternatively, a change in clinical picture automatically equals the reassignment to another, more fitting phenotype, which in turn indicates little to no stability of phenotypes [15]. Recently, Garden et al. tried a new approach to increase both reliability and stability by connecting LCA phenotypes defined at different time points through transition probabilities. Similarly, they found evidence supporting some but not all clinical phenotypes [16]. Gaining in-depth understanding of underlying immune mechanisms of not yet clearly classified groups of children may contribute to a more specific definition.

One facet of defining precise and stable phenotypes is the question of predicting outcomes, i.e., progression of wheeze to childhood asthma and persistence of asthma into adulthood. Longitudinal studies following a cohort from infancy to adulthood are limited; however, it is commonly accepted that the origins of adult asthma at least partially lie in childhood events. For instance, experiencing EVW in childhood appears to be a risk for rapid loss of lung function in adult asthmatics. On the other hand, there are many differences in phenotypes of childhood compared to adult asthma, for example, aspirin-sensitive asthma is a subset rather found in adults, and allergic or eosinophilic and non-allergic or non-eosinophilic asthma are not identical in children and adults. Regarding stability of phenotypes, frequent switching seems to be a phenomenon more common in childhood wheeze and asthma, as the phenotypes of adult asthma appear to be more stable, rather showing variations within one phenotype [17].

Over the past three decades, many birth cohorts have tried to elucidate some of the influences contributing to the development of a certain phenotype. Ongoing efforts to harmonize data from different cohorts will allow comparisons and joint analyses [18]. Efforts, for example, also include genome-wide association studies (GWAS) to identify new candidate genes or whole genome sequencing in small, genetically homogeneous populations to find possible rare gene variants like copy number polymorphisms (CNPs) [19, 20]. Recently, the definition of the term “endotype,” describing a specific pathogenetic mechanism leading to the clinical presentation of asthma, has been brought up. In theory, the identification of new biomarkers and modifiers will lead to a more precise diagnosis and then to individualized phenotypes, enabling a personalized therapy [2]. Potential biomarkers mainly relate to immunological pathways and their regulation either on a transcriptional level, such as micro RNAs (miRNA) [21], or through cytokine regulation [22]. Certain endotypes, such as inflammatory endotypes, have already been established. For instance, the cytokine patterns in induced sputum samples from adult asthmatics significantly differed among clinical phenotypes and correlated with single nucleotide polymorphisms (SNPs) [5, 23].

Intensive efforts to further classify such endotypes especially in children seem to be the most promising approach to further understand the pathogenesis of early-in-life wheeze disorders. This will help to determine whether they are or are not developing into childhood asthma in order to prevent or modify this progression [24]. However, the process of resolving critical challenges as for example the above-described issue of stability of phenotypes could face difficulties and need time, as the required longitudinal continuous measurements of children cohorts are currently still limited.

## Puzzle of novel immune phenotypes

**Fig. 1:**
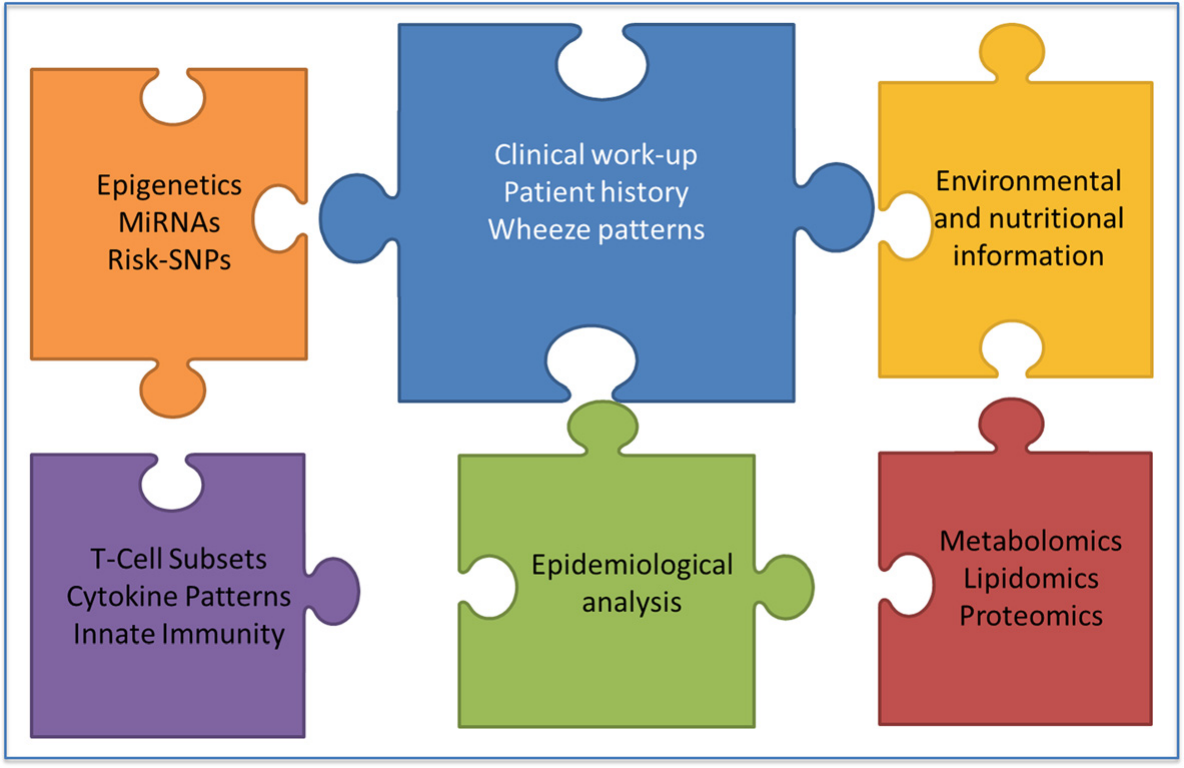
The parts of the puzzle of relevant factors in childhood asthma. Each part and color represents different biological mechanisms and information influencing childhood asthma

**Fig. 2:**
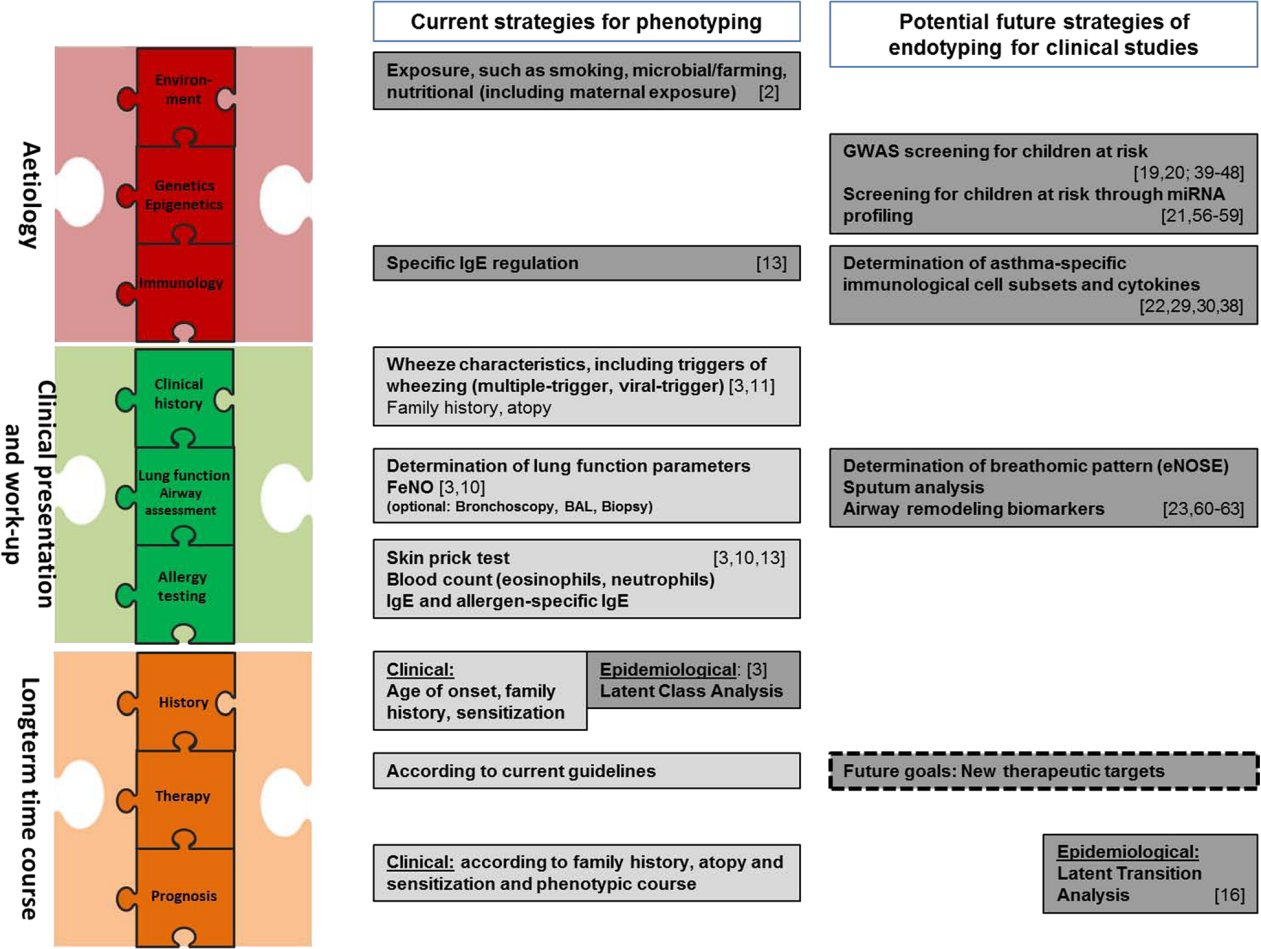
Current and potential future strategies for phenotyping and endotyping, respectively. *Boxes* in *light gray* contain strategies already implemented in clinical practice. *Boxes* in *dark gray* contain strategies currently only used in a research context

The complex puzzle of the development and manifestation of childhood asthma consists of different parts which are illustrated in Figs. 1 and 2 and described in more detail within this chapter.

### Immunological differences in children with asthma

Asthma is a complex disease, and different childhood asthma phenotypes have already been defined by different immunological mechanisms [25]. Several studies aiming to identify endotypes are under way, and their relevance for clinical monitoring and subsequent treatment options is still a subject of discussion. The following chapter will show different approaches to identify immunological patterns and biomarkers for different asthma endotypes in childhood. However, overall biological and immunological mechanisms of asthma are not the main focus of this review and are described in depth elsewhere [2, 26].

Immunological mechanisms in asthma concerning the adaptive immune system have been reduced to the imbalance between Th1 and Th2 CD4+ T cells for many years. However, the shift in favor of Th2 cells cannot explain all features of asthma. In the last years, different T cell subpopulations have emerged with a role in asthma, such as regulatory T cells (Treg) and the recently described Th9 and Th17 cells. Tregs are ascribed immuno-suppressive functions as they induce self-tolerance and suppress allergy and asthma [27]. Even though Treg counts were increased in asthmatic versus healthy children, their numbers did not differ significantly between children with allergic and non-allergic asthma. However, their function does as Tregs of allergic asthmatic children show sufficient suppression of Th1/Th2 cytokines, whereas Tregs from non-allergic asthmatics do not [25]. Th9 cells, which mainly produce IL-9 form another T cell subset that has recently been described to drive asthma development. IL-9 is important for numerous biological functions such as prolonging the survival of mast cells, increasing T cell growth and proliferation and favoring airway remodeling and epithelial mucus production. The sources of IL-9 are different cell types such as Th2 cells, mast cells, and different granulocytes. Murine models of IL-9 overexpression showed pulmonary infiltration, airway inflammation, and bronchial hyperresponsiveness. Administration of anti-IL-9-antibodies for this possible therapeutic target was associated with decreased total lung collagen in a house dust mite (HDM) murine model [28, 29]. In human adult patients with mild to moderate asthma, treatment with anti-IL-9 MEDI-528 showed first promising results [30–32].

IL-17 producing Th cells are accepted as a distinct T cell lineage, as Th17 cells do not develop from Th1 or Th2 cells. They were associated with asthma as increased levels of their pro-inflammatory cytokine IL-17 were found in plasma samples and airway tissue of asthma patients. IL-17 induces the production of pro-inflammatory cytokines and chemokines in epithelial cells, fibroblasts, and neutrophilic granulocytes leading to airway inflammation. IL-17 was also found to play a role in the exacerbation of asthma, which is commonly triggered by viral infections mediated mainly via Toll-like receptor 3 [26, 27]. However, Lunding and colleagues showed in murine experiments that exacerbation of experimental asthma depends on IL-17A which is produced by NK and not Th17 cells [33]. Subsequently, more studies on the role of IL-17 producing cells in different asthma phenotypes and different disease severity are required [30]. Clinical trials using anti-IL-17 treatments in adults have not shown convincing results yet, which may in part be related to the patient selection independent of their underlying pathophysiology [34]. Several links between IL-17 and IL-9 have been identified. They are mediators of both adaptive and innate immune regulation supporting the theory that a close interplay of adaptive and innate immune regulation is critical in asthma.

Most innate immune cell types like monocytes, lung-specific macrophages, dendritic cells (DC), natural killer cells (NK), mast cells, and especially eosinophils are enriched within asthma patients [35]. This diversity shows that asthma is not only driven by allergy. Also, viral infections and bacterial colonization play a role in the severity of asthma, but it is still discussed whether the type of infections and the number of episodes are causally related to, or associated with, asthma or vice versa [36]. Allergic asthmatic children showed specific changes in innate immune regulatory factors such as decreased expression of chloride intracellular channel 4 (CLIC4) and tuberous sclerosis 1 (TSC1) [25]. Children suffering from non-allergic asthma had increased neutrophil counts and IL-1beta secretion and showed an IL-17 shifted immune response upon stimulation of blood cells in vitro. However, the expression of anti-inflammatory proteins like IL-37 and PSTPIP2 was also increased in this phenotype. IL-37 production of stimulated peripheral blood mononuclear cells (PBMCs) was significantly lower in allergic asthmatic children compared to healthy controls. IL-37 actually requires IL-18Rα and SIGIRR/IL-1R8 to diminish allergic airway inflammation in mice. Both IL-37 and IL-18 share the same receptor. IL-37 production in stimulated PBMCs was significantly lower compared to healthy controls [37]. The NLRP3 inflammasome, another target of innate immunity, was shown to be upregulated in neutrophilic asthma and puts the immune system into an activated state with high production of the pro-inflammatory cytokines interleukin (IL)-1b and IL-18 [25, 38, 39].

Furthermore, the recently discovered innate lymphoid cells (ILC) type 2 seem to play a major role in allergic eosinophilic asthma as they secrete asthma-associated cytokines such as IL-13 and IL-5 in response to the epithelia derived cytokines IL-25, IL-33, and thymic stromal lymphopoietin (TSLP) [22].

### Genetic and epigenetic influences in asthma

Transcription and translation of different genes are regulated in a very complex manner by different meshing regulation strategies and are additionally influenced by gene variants between individuals and populations. Cellular regulatory mechanisms are, among others, epigenetic histone acetylation, DNA methylation, and posttranscriptional regulation via miRNA. Together, they are likely mechanisms and currently a primary focus of research studies, which may influence the different asthma phenotypes in children and in adults.

In GWAS, several new loci were identified, which are associated with childhood asthma. This involves for instance the ORMDL [40, 41], TSLP [42–44], IL-33 [45, 46] TLR, and HLA-C [47] gene families. Recent studies showed that gene variants are not only associated with asthma occurrence but also with clinical development and severity. For example, children with gene variants in STAT4, JAK2, MX1, VDR, DDX58, EIF2AK2 [48], and cadherin-related family member 3 (CDHR3) [49] showed a higher incidence of viral induced exacerbations. Another GWAS study identified 11 gene variants associated with hay fever in combination with the asthma phenotype [50] which might represent the allergic asthma phenotype. Yet, genetics can only partly explain heritability of childhood asthma [45, 51].

Besides genetics, the very complex field of epigenetic mechanisms seems to play an important role in the development of asthma. Especially, prenatal and early childhood DNA methylation and histone modification may be useful targets as new biomarkers for the development and/or manifestation of asthma. Various lifestyle factors were shown to influence epigenetic regulation of individuals such as smoking, obesity, fish oil consumption, and microbe exposure during pregnancy as recently summarized [52, 53]. Maternal exposure to microbes for example was shown to protect the offspring from asthma in mice. Brand et al. have accounted this effect to the protection from the loss of histone 4 acetylation on the IFNγ promotor of CD4 T cells in the offspring. Not only protective effects were driven by epigenetic mechanisms. Also, FOXP3 and IL-13 histone acetylation was increased and positively correlated with higher protein level of IL-13 in allergic asthmatic children [54]. To study epigenetic modifications, blood leucocytes and lymphocyte subtypes are possible biomaterials in pediatrics [55–57] with the advantage of availability and ethical feasibility as compared to lung biopsy tissue. Studies combining genetic and epigenetic targets are under way to disentangle their relevance in childhood asthma phenotypes.

In the field of posttranscriptional regulation in gene expression, one mechanism is mediated by small non-coding RNA sequences. These miRNAs can bind complementary to messenger RNA (mRNA) and lead to a repression of protein synthesis through degradation of mRNA or transcription blockade. They are gaining in importance as both potential biomarkers and therapeutic targets. Specific miRNA sequences have already been shown to be differentially expressed in various diseases. The role of miRNA in asthma and other respiratory diseases were summarized in the recently published mini review of Maltby et al. [58]. Current knowledge is based on screening of different patient materials and cell types, mainly in adult patients. Bronchial epithelial cell miRNA expression differed in more than 60 miRNA, whereas miRNA from bronchial alveolar lavage fluid (BALF) differed in 24 instances. Seven differently expressed miRNA (miR-192, miR-24, miR-26a, let7a, let-7d, miR-221, and miR-485-3p) were described in peripheral blood samples. T cell-specific differences were found in miR19a [58]. A recent study found two miRNA expression profiles associated with high or low eosinophil count respectively. They also reported that the levels of several circulating miRNAs (miR-125b, miR-16, miR-299-5p, miR-126, miR-206, and miR-133b) were able to predict the allergic/asthmatic status in adults. As circulating miRNAs are easily accessible, they could possibly be used as relatively non-invasive biomarkers [59]. Another study notably found miRNAs (miR-223-3p, miR-142-3p, and miR-629-3p) that are elevated in patients with severe asthma, where endotyping is most needed. As they were expressed in bronchial epithelium and neutrophils, these findings possibly even suggest a pathophysiological contribution to this neutrophilic inflammatory endotype [60]. In another study, miRNA-21 serum levels were found to be increased in asthmatic compared to healthy children. This may also be helpful for further discrimination of asthma phenotypes, as this miRNA was higher expressed in steroid-resistant compared to steroid-sensitive patients. As potential target of miRNA-21, the authors identified IL-12p35 [61]. Other pediatric studies identified circulating miR-3162-3p, miR-1260a [62] and mi497, let-7e, and miR-98 as significantly different expressed [21] in asthmatics compared to healthy controls.

For future studies, specific miRNA patterns could be used to identify children at risk for asthma or for discrimination of asthma phenotypes, as the required plasma samples are easily accessible. Yet, several facets of differences and similarities in miRNA expression in children and adults require further studies: miRNA stability over time needs to be investigated. Further, the prerequisite for a screening is a clear discrimination between healthy controls and patients. Also, the specificity of miRNA for asthma needs to be validated as the same miRNA might be differentially regulated in different lung diseases. In the review of Deshpande et al., the preclinical achievements of targeting miRNA were discussed. However, the problem of the delivery mechanisms of miRNA still has to be solved [63]. In summary, miRNA may (i) serve as a promising tool for the diagnosis of asthma and distinct phenotypes at an early age and (ii) be further evaluated as therapeutic option.

Taken together, epigenetic modifications, combined with functional transcriptional mechanisms, are promising candidates contributing to close the existing gap of knowledge of the molecular mechanisms of asthma and also allergy, in particular for different phenotypes. Yet, the practical application for clinics is still theory, and ongoing and future studies would profit from implementation of direct use for asthma patients to truly impact on asthma diagnosis or treatment.

### Metabolomics in asthma

Verification of cellular metabolic products in different body fluids represents a novel approach to identify patients with asthma. The detection and specification of such metabolic molecules have been studied with mass spectrometry (MS) and nuclear magnetic resonance (NMR) spectroscopy in terms of lipids and glucose metabolites. Research focused on airway biofluids of the lung [64]. Yet, to date, no metabolic molecule was shown to be specific for asthma and is in clinical use. Another non-invasive bio fluid characteristic for metabolic changes may be urine, which was tested in different studies to differentiate asthma patients from healthy adults. Wedes et al. identified increased bromotyrosine in asthma patients, which was (i) associated with eosinophils and (ii) with a higher risk of exacerbation in this study [65]. A different promising metabolomics research field is investigation of breathomics, i.e., the detection of volatile organic compounds (VOC) in breath with the so-called eNose. The breath consists of multiple VOC, and to date, several thousands were identified which represents only the tip of the iceberg [66]. Differences of exhaled patterns were shown in children with wheeze and pulmonary infection with rhinovirus, indicating that eNose profiles can be a tool to predict asthma in wheezing children [67]. Identification and prediction of asthma and different asthma endotypes with the help of exhaled metabolites seems to be promising, in particular for children, as the method is non-invasive and analyzes metabolomic patterns rather than focusing on one single biomarker. Even so, a controlled and standardized breathomic analysis seems not easily practicable for small children, and thus, urine sampling may be an alternative method of choice if shown specific. Nevertheless, metabolic research is promising, and further research will help to disentangle the underlying immunological and metabolic mechanisms and to identify involved molecules.

## Implications and outlook

In summary, multiple studies indicate that the use of endotyping may help to predict a better response to asthma treatment and potentially novel therapies. Also, there is increasing evidence that selecting patients based on biomarkers can identify subgroups that are likely to respond to biological treatments [24, 68]. For pediatric studies, these data are just starting to emerge due to technical and ethical issues. However, although biomarkers are not implemented in daily pediatric practice yet, they may be a very valuable tool in the future to reach the goal of prevention [68, 69]. In the recent review of Berry et al., blood eosinophils and FENO were suggested as biomarkers to predict an effective administration of steroids allowing individualized treatment [68].

Additionally, recent studies about anti-IL-5-treatments have successfully used eosinophil count to select patients. In children, non-invasive transcriptome analysis in sputum gene expression analysis have allowed to form three clusters of near-fatal, severe, and mild asthma in children (and adults) [70]. However, the relevance for personalized medicine still needs to be shown. Also, transcriptomics can discriminate subtypes of asthma and may have a role in delivery of individualized therapy [71]. In pediatric patients, nasal brushing may be of use as it is a more easily accessible surrogate for transcriptomics than induced sputum, and of special relevance in TH2-low asthma endotypes. Regarding the combination of different methods for endotyping, a recently published article of McGeachie et al. [72] featured combined metabolomics data measured in the plasma of 20 asthma patients with genome-wide genotype information, gene expression, and methylation data from the Asthma BRIDGE cohort [73]. This complex metabolomics pathway and network analysis enabled the researchers to identify the importance of the arachidonic acid and linoleic acid metabolism involved in asthma control and the prediction of uncontrolled asthma. They identified an altered sphingolipid metabolism in uncontrolled asthma which is associated with the cellular response to albuterol. The combination of available data from interdisciplinary research teams like the current U-BIOPRED cohort [74] combining expertise from medicine, biology, epidemiology, and mathematics are critical to disentangle the multifaceted interplay, in particular for distinct asthma phenotypes. Immunobiological tools to differentiate between different asthma phenotypes are available and will be improved by using the novel markers in clinical differentiation in a cost-effective and practical way. The exact definition of asthma and asthma phenotypes in early and later childhood is the prerequisite for its successful and possibly more specific treatment. Childhood wheezing and asthma are clearly associated with decreased lung function and chronic obstructive pulmonary disease (COPD) in older age [75]. The asthma-COPD overlap syndrome is associated with clinical symptoms of both diseases which may lead to insufficient diagnosis and therapy of these patients [76]. It shows that asthma is a life course disease with many faces often starting in childhood.

Current differentiation and classification of phenotypes in particular in young asthma patients will profit from future interdisciplinary research networks which, if successful, will result in identification of subgroups with distinct endotypes subsequently profiting from individualized medicine with decreased side effects. Although novel findings will lead to an increase of parts of the puzzle, these parts have to be combined leading to a more distinct definition of few clearly defined phenotypes.

There may be several combinations of molecular analysis imaginable to differentiate distinct phenotypes (Fig. 1). One possibility is grouping based on a number of components, including clinical phenotyping in combination with immunological and epigenetic markers. However, one vision is to identify a mixture of single or several components out of different biological levels (e.g., a combination of the most relevant genotypes), a selection of epigenetic modification in addition to a couple of miRNAs leading to changes in immune regulation, and environmental influences to best define a clinical phenotype. Whether this distinction of phenotypes can be also applied for the prediction of the course of disease and specific therapies needs to be carefully evaluated. In analogy, all possible biomarkers need to be carefully validated in cohort studies [77]. Additionally, continued efforts in endotyping will lead to a number of questions concerning the implementation in clinical care as described in a recent review by Galli, e.g., the question of who will, based on what evidence, decide which endotypes should be generally accepted [24].

For children and their families, a better prediction of the course of the disease will also contribute to an increased quality of life. Reduction of asthma-related personal and societal burden is achievable within the next decade through the development of novel preventive and possible therapeutic regimens. Figure 2 illustrates currently used and possible future strategies for phenotyping and endotyping. Future studies need to determine if available omics and their combinations have the capacity to truly identify patients at risk of adverse outcomes (such as hospitalization) early in life, to personalize management, improve outcomes, and enhance selection for current and emerging treatment strategies or will be rather useful for advancements in the pathogenesis of asthma.

## Conclusion

In summary, the definition of exact asthma phenotypes especially in children is complex. Clinical classification depending on current symptoms does not reflect the heterogeneity and course or stability of the disease and might lead to insufficient treatment. Therefore, the definition of asthma and its phenotypes depending on endotypes including immunological and genetic mechanisms are needed for potentially more effective and tailored therapy in the long term. Developing an “asthma phenotype index” including clinical and molecular criteria of several facets relevant and stable over time would facilitate practical use in clinics, if positive and negative predictive values are superior to current management. In order to truly have an impact on a more specific asthma diagnosis, modulated treatment and patient’s recommendation, these challenges have to be approached in the next decade of asthma research.

## Abbreviations

BALF, bronchial alveolar lavage fluid; CNPs, copy number polymorphisms; COPD, chronic obstructive pulmonary disease; DC, dendritic cell; EVW, episodic viral wheeze; GWAS, genome-wide association study; HDM, house dust mite; IL, interleukin; ILC, innate lymphoid cells; LCA, latent class analysis; miRNA, micro RNA; mRNA, messenger RNA; MTW, multiple-trigger wheeze; NK, natural killer cell; SNPs, single nucleotide polymorphisms; Th, T helper cell; Tregs, regulatory T cells; VOC, volatile organic compounds
